# Editorial: Structure and mechanism of microbial membrane active transporters

**DOI:** 10.3389/fmicb.2025.1741179

**Published:** 2025-11-21

**Authors:** Xiaoxu Jiang, Chuck R. Smallwood, Phillip E. Klebba, Maria Carmela Bonaccorsi di Patti

**Affiliations:** 1 Department of Chemistry and Biochemistry, California State University, San Bernardino, CA, United States; 2 Special Projects Department, Sandia National Laboratories, Albuquerque, NM, United States; 3 Department of Biochemistry and Molecular Biophysics, Kansas State University, Manhattan, KS, United States; 4 Department of Biochemical Sciences ‘A. Rossi Fanelli', Sapienza University of Rome, Rome, Italy

**Keywords:** membrane transporter, membrane transport, membrane protein, structure-function, mechanism

Membrane active transporters play essential roles in microbial physiology. They couple energy transduction to conformational changes that drive translocation of nutrients, substrates and ions, as well as molecular communication. The structure and function of microbial membrane active transporters are highly diverse. Typical examples include the primary active transporters in the ATP-binding cassette (ABC) superfamily (Thomas and Tampé, 2020; Davidson et al., 2008; Locher et al., 2002), the secondary active transporters in the Major Facilitator Superfamily (MFS) (Drew et al., 2021; Kaback and Guan, 2019), and the ligand-gated porins in the TonB-dependent transporter (TBDT) family (Klebba et al., 2021). As structural, proteogenomic, and computational methods advance, active transporters are increasingly recognized as dynamic molecular machines whose mechanisms can now be visualized and modeled with remarkable precision, building on decades of biochemical and biophysical discovery that established the foundations of this field. The transporter studies recruited in this Research Topic provide us with new insights into the field including structure-function of sugar transporters in yeast, structural prediction and classification of ABC complexes in *Bacillus subtilis*, Type VI Secretion System (T6SS) in *Bacteroides fragilis*, amino acids uptake in *Escherichia coli* and bacterial spore germination.

The process of xylose uptake in yeast is critical for the yeast-based biofuel production. In this Research Topic, Taveira et al. present a comprehensive review of MFS and SWEET xylose transporters, integrating crystallography, cryo-EM, and molecular dynamics to reveal how rocker-switch motions and gating kinetics determine substrate specificity. By pairing structural data with machine-learning-guided sequence analysis and directed evolution, the authors identify conserved features that underlie transport efficiency and regulatory diversity. Their synthesis illustrates how iterative cycles of modeling and experimentation can illuminate long-standing questions in sugar transport, from substrate coupling to allosteric control.

Artificial Intelligence (AI) technology is reshaping the field of structural biology by allowing researchers to quickly and accurately predict three-dimensional protein structures. This is particularly meaningful for the structure determination of membrane transporters, which remains challenging using traditional experimental approaches. Using AlphaFold2-Multimer, Mahendran and Orlando performed structural predictions of all potential ABC transporter complexes encoded in the genome of *B. subtilis*, providing not only a comprehensive structural gallery of ABC transporters in this model organism but also suggesting novel folds and topologies not observed in previously solved structures. Comparisons between the predicted architecture and experimentally determined structures indicate that AlphaFold harnesses high accuracy and reliability. Their study demonstrates the enormous potential of AI in the structural determination of membrane proteins. Compared to X-ray crystallography and cryo-EM, AI offers significant advantages in both efficiency and cost-effectiveness. On the other hand, the authors also point out that some predicted structures contain noticeable errors, suggesting that AI algorithms could be further improved, particularly in considering the effects of protein–lipid interactions on transporter structure. Therefore, experimental methods are still needed for data confirmation.

In another endeavor, Zakharzhevskaya et al. uncover a vesicle-associated Type VI Secretion System (T6SS) in *B. fragilis*, showing that its structural and regulatory components remain active even without direct cell contact. Proteogenomic mapping identifies effector–immunity pairs within outer-membrane vesicles, suggesting a dual mode of energy-dependent secretion and passive dissemination. This finding expands the mechanistic scope of T6SS, linking molecular architecture to intercellular communication and competitive dynamics.

The acquisition of nutrients is one of the defining properties of biological membranes. Bacteria acquire amino acids through two main mechanisms: synthesizing them from simpler precursors or absorbing them directly from their environment. Although prototrophic strains can produce all 20 standard amino acids *de novo*, when available bacteria will preferentially obtain them from the environment, because the latter route is more energy-efficient than internally synthesizing them. They do so with active transport systems for specific free amino acids, that encompass well-known primary or secondary transporters in the inner or cytoplasmic membranes of Gram-negative bacterial cells. But what is the cellular response when the main uptake systems for a particular amino acid are lost? Bubnov et al.*, “Multiple routes for non-physiological L-threonine uptake in Escherichia coli K-12*,” explores and answers this question, by finding multicopy suppressors and chromosomal mutations that complement the loss of L-threonine uptake activity. In an *E. coli* strain lacking the major threonine-specific permeases, their phenotypic assays identified YhjE and SdaC as two alternative entry points for threonine. These data both illustrate the multiple redundant mechanisms for absorption of these essential solutes, as well as the potential complexity of engineering commercial strains for threonine production.

The study of membrane protein structure-function relationships has hugely benefited from the combination of computational methods and site-directed mutagenesis, the latter allowing to experimentally test new hypotheses provided by the former. A good example is the paper contributed by Chen et al. on GerAB, one of the subunits of the *B. subtilis* spore germinant receptor GerA. This receptor is part of the germinosome, a complex that resides at the inner membrane and responds to nutrients to start *B. subtilis* spore germination. Employing Steered Molecular Dynamics (SMD), the authors investigate the properties of a previously predicted water channel in GerAB and identify three residues (Y97, L199 and F342) that may interfere with water passage. Site-directed mutagenesis reveals a role for these residues in the stability of GerAB; in fact, the mutant proteins are expressed at much lower level than the wild type, compromising the function of the germinant receptor. Although this result does not allow to assign a specific function of Y97, L199, and F342 in water passage, it illustrates how unanticipated roles of amino acid residues beyond those predicted by computational methods may be uncovered when experimental methods come into play.

Together, the studies in this Research Topic underscore how mechanistic biochemistry, anchored in structure, dynamics, and regulation, continues to define and expand our understanding of microbial membrane transport. We expect that the results presented here will also shed light on other Research Topics regarding membrane proteins and membrane transport.

## References

[B1] Davidson A. L. Dassa E. Orelle C. Chen J. (2008). Structure, function, and evolution of bacterial ATP-binding cassette systems. Microbiol. Mol. Biol. Rev. 72, 317–364. doi: 10.1128/MMBR.00031-07 18535149 PMC2415747

[B2] Drew D. North R. A. Nagarathinam K. Tanabe M. (2021). Structures and general transport mechanisms by the major facilitator superfamily (MFS). Chem. Rev. 121, 5289–5335. doi: 10.1021/acs.chemrev.0c00983 33886296 PMC8154325

[B3] Kaback H. R. Guan L. (2019). It takes two to tango: the dance of the permease. J. Gen. Physiol. 151, 878–886. doi: 10.1085/jgp.201912377 31147449 PMC6605686

[B4] Klebba P. E. Newton S. M. C. Six D. A. Kumar A. Yang T. Nairn B. L. . (2021). Iron acquisition systems of gram-negative bacterial pathogens define tonb-dependent pathways to novel antibiotics. Chem. Rev. 121, 5193–5239. doi: 10.1021/acs.chemrev.0c01005 33724814 PMC8687107

[B5] Locher K. P. Lee A. T. Rees D. C. (2002). The *E. coli* BtuCD structure: a framework for ABC transporter architecture and mechanism. Science 296, 1091–1098. doi: 10.1126/science.1071142 12004122

[B6] Thomas C. Tampé R. (2020). Structural and Mechanistic Principles of ABC Transporters. Annu. Rev. Biochem. 89, 605–636. doi: 10.1146/annurev-biochem-011520-105201 32569521

